# The Diverse Roles of the Histone‐Like Nucleoid Structuring (H‐NS) Protein in *Vibrio parahaemolyticus*

**DOI:** 10.1155/genr/6503207

**Published:** 2026-02-16

**Authors:** Hao Tang, Yiquan Zhang

**Affiliations:** ^1^ Department of Clinical Laboratory, Affiliated People’s Hospital of Jiangsu University, Zhenjiang, 212001, Jiangsu, China, ujs.edu.cn; ^2^ Department of Clinical Laboratory, Nantong Third People’s Hospital, Affiliated Nantong Hospital 3 of Nantong University, Nantong, 226006, Jiangsu, China

**Keywords:** biofilm, H-NS, motility, quorum sensing, regulation, *Vibrio parahaemolyticus*, virulence

## Abstract

The histone‐like nucleoid structuring (H‐NS) protein is a global transcriptional regulator critical for bacterial genome organization and gene expression. In *Vibrio parahaemolyticus*, H‐NS represses virulence factors such as thermostable direct hemolysin (TDH), type III secretion systems (T3SS1/T3SS2), and type VI secretion systems (T6SS1/T6SS2). It also modulates swimming motility by activating polar flagellar genes while repressing swarming motility and lateral flagellar systems. Additionally, H‐NS influences biofilm formation through regulation of exopolysaccharide synthesis and cyclic di‐GMP (c‐di‐GMP) metabolism. Emerging evidence suggests cross‐regulation with quorum sensing (QS) systems, though direct mechanistic insights into *V. parahaemolyticus* remain sparse. Furthermore, a recent study indicates H‐NS roles extend to osmotic stress adaptation, such as regulating the ectoine biosynthetic pathway. This review synthesizes current knowledge on H‐NS‐mediated regulation in *V. parahaemolyticus* and offers new insights for future research.

## 1. Introduction

*Vibrio parahaemolyticus* is a gram‐negative, halophilic bacterium widely distributed in seawater, marine sediments, and marine animals such as fish and shellfish [1]. Its pathogenicity is closely linked to the expression of virulence determinants, including thermostable direct hemolysin (TDH), type III secretion systems (T3SS1/T3SS2), and type VI secretion systems (T6SS1/T6SS2) [2]. These factors exhibit distinct physiological activities and contribute uniquely to pathogenesis [2]. Additionally, *V. parahaemolyticus* demonstrates robust biofilm‐forming capabilities [3]. Biofilm‐associated *V. parahaemolyticus* exhibits heightened resistance to adverse growth conditions compared to planktonic cells [4]. Biofilms also enhance bacterial adhesion to host surfaces, immune evasion, and pathogenicity by modulating virulence gene expression [5, 6]. The pathogenicity and biofilm formation of *V. parahaemolyticus* are tightly regulated by cyclic di‐GMP (c‐di‐GMP) and its associated regulatory network [7]. c‐di‐GMP is generated by diguanylate cyclase containing a GGDEF domain and degraded by phosphodiesterase with a EAL or HD‐GYP domain [7]. Elevated c‐di‐GMP levels promote biofilm formation while repressing virulence factor expression and motility [7]. Notably, the expression of these and many other phenotypes is globally coordinated by the quorum sensing (QS) system, a cell‐density‐dependent gene regulatory network [8].

The histone‐like nucleoid structuring (H‐NS) protein is a highly abundant DNA‐binding protein in enteric bacteria that preferentially binds AT‐rich and highly curved DNA, subsequently oligomerizing across adjacent AT‐rich regions [9]. H‐NS plays diverse functions in bacterial cells, including modulating DNA folding, facilitating genome evolution, and globally regulating gene expression [9–11]. Initially characterized as a transcriptional repressor of horizontally acquired AT‐rich genes [10], H‐NS has since been shown to positively control gene expression at both transcriptional and posttranscriptional levels [12–16]. Its regulatory targets span critical cellular pathways such as environmental adaptation, biofilm formation, virulence, and metabolism [17]. While the roles of H‐NS in *V. parahaemolyticus* have been extensively studied, key questions remain unresolved. This review synthesizes current knowledge on H‐NS‐mediated regulation, with a particular focus on its interplay with the QS system and its subsequent effects on motility, virulence, and biofilm formation in *V. parahaemolyticus*, while identifying critical gaps for future investigation.

## 2. Structure of H‐NS Protein

The polypeptide chain of *E. coli* H‐NS comprises 137 amino acid residues organized into two independent domains: an N‐terminal oligomerization domain (NTD, residues 1–83) and a C‐terminal DNA‐binding domain (CTD, residues 91–137) (Figure 1(a)) [11]. The NTD contains four α‐helices (H1, H2, H3, and H4), while the CTD consists of two β‐sheets (β1 and β2) and two α‐helices (H5 and H6) (Figure 1(b)) [11]. These domains are linked by a flexible linker. H‐NS undergoes high‐order self‐association via two distinct forms: head‐to‐head and tail‐to‐tail dimerization (Figures 1(c) and 1(d)) [11]. Head‐to‐head dimers are stabilized by interactions between helices H1 and H2, whereas tail‐to‐tail interactions involve helices H3 and H4 forming a helix‐turn‐helix motif [11]. This motif interlocks the C‐termini of the two protomers in an antiparallel orientation [11]. The helix‐turn‐helix is followed by the CTD, which includes β1, β2, H5, and H6 [11, 20]. H‐NS employs a conserved DNA‐binding mechanism where a short loop between β2 and H5, containing a ‘QGR’ motif, selectively interacts with the minor groove of DNA strands [11, 20]. These sequences are preferentially targeted due to their narrower minor grooves, which enhance binding affinity. Protein sequence comparisons reveal that H‐NS residues are not fully conserved between *E. coli* and *V. parahaemolyticus* (Figure 1(a)). However, the presence of a conserved QGR motif in *V. parahaemolyticus* H‐NS suggests that it likely shares the same DNA‐binding mechanism as its *E. coli* counterpart (Figure 1(a)).

FIGURE 1Schematic representation of H‐NS domain organization and dimerization modes. Structure and conservation of H‐NS: Sequence alignment of orthologs in *E. coli* and *V. parahaemolyticus* (a) and the domain organization underpinning its oligomeric assembly (b). Sequences were derived from *V. parahaemolyticus* RIMD 2210633 [18] and *E. coli* [19]. Identical residues are marked with asterisk (∗). H, *α*‐helix; β, β‐strands. The residues forming α‐helices or β‐strands were labeled with shadows. NTD residues are highlighted in blue; CTD residues are in in purple. The conserved sequence (QGR) is marked with a red box. H‐NS oligomerizes through sequential head‐to‐head (c) and tail‐to‐tail (d) interactions.

**Figure:**
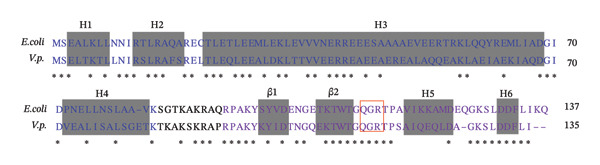
(a)

**Figure:**
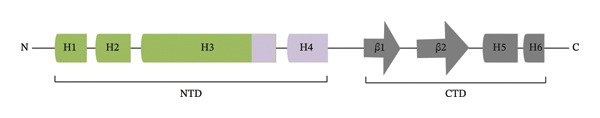
(b)

**Figure:**
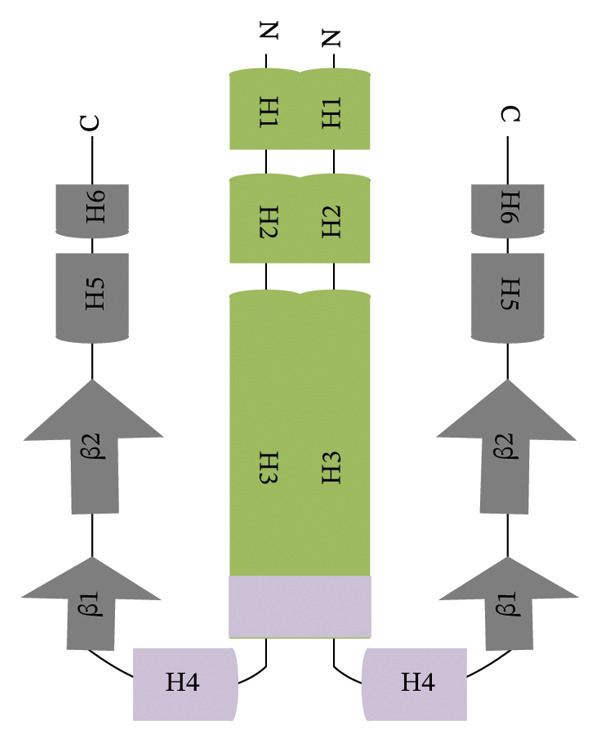
(c)

**Figure:**
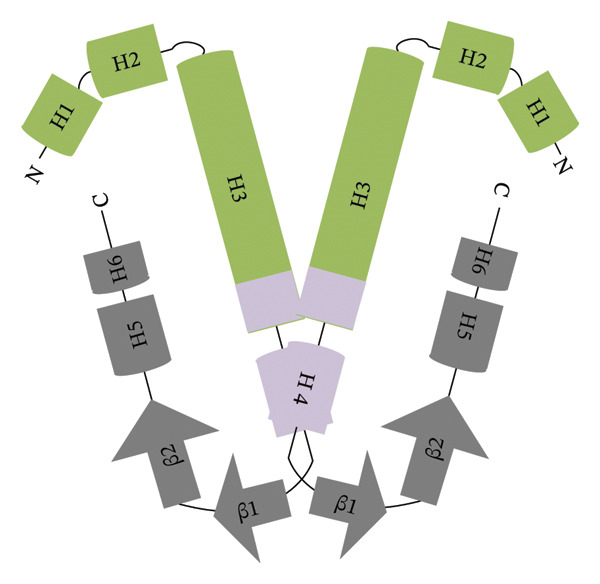
(d)

### 2.1. The QS System in *V. parahaemolyticus*

QS is a cell density–dependent regulatory mechanism that allows bacteria to coordinate collective behaviors, including virulence, biofilm formation, motility, and stress adaptation [21]. In *V. parahaemolyticus*, the QS circuit revolves around two key master regulators: AphA and OpaR, which function reciprocally at low‐cell density (LCD) and high‐cell density (HCD), respectively [22, 23]. At LCD, low autoinducer (AI) levels lead to phosphorylated LuxO, which activates the expression of Qrr small RNAs [22]. These Qrr sRNAs posttranscriptionally activate *aphA* expression while repressing *opaR* [22]. AphA then activates the expression of genes related to virulence, motility, and biofilm formation, while repressing its own expression and that of opaR [23]. Conversely, at HCD, high AI levels cause LuxO dephosphorylation, reducing Qrr sRNA production [22, 23]. This relieves the repression on *opaR*, allowing its high expression [23]. OpaR then activates *qrr* genes while repressing *aphA*, virulence genes, flagellar genes, and biofilm‐related genes [23]. Thus, the QS system establishes a sophisticated switch that globally reprograms gene expression in response to population density, directly controlling the major phenotypic outcomes discussed in this review.

### 2.2. Interplay Between H‐NS and the QS System

Emerging evidence suggests a significant regulatory interplay between H‐NS and the QS system in *Vibrio* species, positioning H‐NS as a key modulator within this global regulatory network [13, 24–27]. For example, in *V. fischeri*, the *lux* system is encoded by two divergently transcribed adjacent operons sharing a common regulatory region [24–26]. One operon contains seven genes (*luxICDABEG*) encoding enzymes required for AI synthesis, while another operon contains a single gene encoding the QS regulator LuxR [24–26]. Transcription of both *luxR* and *luxICDABEG* is negatively controlled by H‐NS, whereas *luxICDABE* transcription is positive regulated by LuxR [24–26]. In *V. harveyi*, H‐NS and LuxR coregulate 28 promoters controlling 63 genes genome‐wide; LuxR displaces H‐NS from promoter DNA, functioning as an antisilencer at H‐NS‐repressed QS loci by disrupting H‐NS nucleoprotein complexes [27]. In *V. parahaemolyticus*, H‐NS indirectly activates transcription of *opaR* (a *luxR* homolog) but exerts no regulatory effect on *aphA* transcription [13]. This interaction suggests that H‐NS can influence the QS regulatory hierarchy, potentially modulating the transition between LCD and HCD states and the subsequent expression of QS‐controlled phenotypes like virulence, biofilm, and motility. However, direct experimental evidence linking H‐NS and QS in *V. parahaemolyticus* remains limited, underscoring the need for mechanistic studies to elucidate their functional synergies in gene expression regulation.

### 2.3. Regulation of Virulence Gene Expression

*V. parahaemolyticus*, the leading cause of seafood‐associated gastroenteritis worldwide, produces multiple virulence determinants, including TDH, T3SS1, T3SS2, T6SS1, and T6SS2 [28]. The expression of these virulence factors is under complex control involving both specific regulators and global systems like QS [2, 22]. TDH contributes to hemolytic activity and partially mediates cytotoxicity and enterotoxicity [28]. T3SS1 primarily drives hose cell cytotoxicity, whereas T3SS2 is critical for enterotoxicity, as demonstrated in a rabbit ileal loop model [28, 29]. Besides mediating enterotoxicity, T3SS2 also promotes intracellular invasion and TDH secretion [30, 31]. Studies have shown that T3SS2 effector proteins such as VopC and VopL disrupt host cytoskeletal reorganization, facilitating bacterial internalization and intracellular escape, thereby enhancing their survival and dissemination within host cells [30]. Similarly, T6SS1 is involved in adhesion and antibacterial activities, while T6SS2 plays a major role in cell adhesion and biofilm formation [32–34].

**FIGURE 2:**
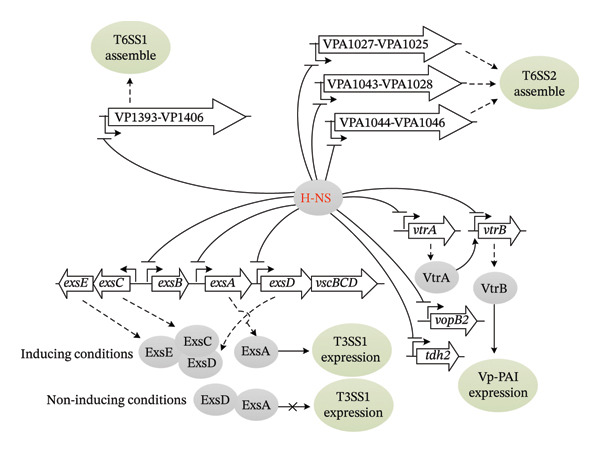
Regulation of major virulence genes by H‐NS in *V. parahaemolyticus*. Arrows represent positive regulation. Flat line ends indicate negative regulation. Broken‐line arrows represent gene promoters. Cross denotes inhibition of the regulatory pathway. Dashed arrows signify translation or a complex positive correlation process.

ExsACDE is the key regulatory system of T3SS1, and the genes encoding this system are located within the T3SS1 genetic cluster (VP1656‐VP1702) [35]. ExsA, a AraC/XylS family transcriptional regulator, activates T3SS1 gene expression under inducing conditions (such as contact with host cells) [36]. Specifically, ExsA binds to the promoter region of T3SS1 genes, promoting their expression and facilitating the assembly and function of the T3SS [37]. Under noninducing conditions, ExsD forms a complex with ExsA, preventing its binding to the T3SS1 promoter and thereby inhibiting T3SS gene expression [36]. ExsC, a T3SS chaperone protein, counteracts this inhibition by binding to ExsD [36]. This interaction relieves ExsA repression, allowing it to activate T3SS genes. Additionally, ExsE, secreted extracellularly via T3SS1 under inducing conditions, enhances ExsC‐ExsD binding, further modulating ExsA activity and T3SS1 gene expression [38]. An earlier research suggested that H‐NS could modulate T3SS1 by repressing *exsA* expression [39]. Subsequent studies revealed that H‐NS directly binds to the promoter–proximal regions of the *exsAD-vscBCDFGHIJKL*, *exsBAD-vscBCDFGHIJKL*, and *exsD-vscBCDFGHIJKL* operons, inhibiting their transcription (Figure 2) [40, 41]. This inhibition represses the expression of ExsA and ExsD. Since *exsC* and *exsB* share an intergenic region and are transcribed in opposite directions [42], H‐NS binding to the *exsBAD-vscBCDFGHIJKL* promoter likely affects *exsC* transcription. Additionally, *exsC* and *exsE* are cotranscribed [42]. Therefore, H‐NS directly regulates the expression of ExsACDE system to modulate T3SS1 activity. However, whether H‐NS directly inhibits *exsCE* transcription or regulates other operons within the T3SS1 genetic clusters remains to be investigated further.

Two *tdh* genes (*tdh1* and *tdh2*) and a group of genes encoding T3SS2 are located on an 80 kb pathogenicity island (Vp‐PAI; VPA1312‐VPA1398) on the smaller chromosome [35]. VtrA and VtrB, two ToxR‐type transcriptional regulators encoded by Vp‐PAI, control the expression of Vp‐PAI genes, including those for TDH and T3SS2 [43]. VtrC, a co‐component of the VtrA/VtrC signal transduction system, binds bile acids to activate the DNA‐binding domain in VtrA, which subsequently induces *vtrB* transcription [44, 45]. A positive autoregulatory loop governing *vtrB* transcription, driven by read‐through transcription from the upstream operon, is critical for regulating T3SS2 and the virulence it mediates in *V. parahaemolyticus* [46]. H‐NS serves as a thermal and salt switch with sensory and regulatory properties, regulating T3SS2 gene expression by directly repressing *vtrB* expression in *V. parahaemolyticus* [47]. H‐NS is also able to bind to the promoter–proximal region of *vtrA* to repress its transcription [41]. Furthermore, H‐NS directly suppresses the transcription of *vopB2* and *tdh2* [41]. In summary, H‐NS regulates the expression of Vp‐PAI genes by modulating VtrA and VtrB expression, and it directly represses the promoters of several other genes within this pathogenicity island, such as *vopB2* and *tdh2* (Figure 2). However, it remains unknown whether there is an antagonistic relationship between H‐NS and VtrA and/or VtrB, and how they coordinately regulate Vp‐PAI gene expression under different environmental conditions. Although H‐NS binding sites have been characterized within the promoter regions of *vtrA*, *vtrB*, *vopB2*, and *tdh2* [41, 47], the complete landscape of H‐NS binding across the Vp‐PAI and its full repertoire of direct target genes remain to be fully mapped.

The T6SS1 genetic cluster (VP1386–VP1420) comprises 34 coding genes organized into at least 7 operons including VP1393–VP1406 [22]. The T6SS2 genetic cluster contains 22 genes grouped into 3 operons: VPA1027–VPA1025, VPA1043–VPA1028, and VPA1044–VPA1046 [22]. H‐NS serves as a negative regulator of T6SS1 under low‐salt conditions or in the absence of surface‐sensing activation; however, surface‐sensing activation or high‐salt conditions alleviate H‐NS‐mediated repression [48]. H‐NS directly represses the transcription of the VP1393–VP1406 operon by binding to its promoter DNA region [49]. Furthermore, H‐NS directly represses all three operons in the T6SS2 genetic cluster (Figure 2) [41]. However, the expression of T6SS1 and T6SS2 is tightly regulated by a complex regulatory network (e.g., QS, QsvR, and ToxR) and environmental conditions (e.g., temperature and salinity changes) [22]. It remains unclear whether H‐NS interacts with other regulatory proteins to coordinately regulate T6SS expression or whether its regulatory mode dynamically adapts to varying environmental conditions.

### 2.4. Regulation of Flagellar Gene Expression and Motility

Bacterial motility in liquid or across moist surfaces can be categorized into swimming, swarming, twitching, gliding, and floating; of these, only swimming and swarming are flagella‐dependent [50, 51]. *V. parahaemolyticus* uniquely employs dual flagellar systems: a single polar flagellum and peritrichous lateral flagella [52]. The polar flagellum drives swimming in liquid environments, while the lateral flagella mediate swarming on surfaces or in viscous liquids [52]. Notably, the expression of flagellar genes is also subject to QS control, with AphA activating and OpaR repressing their expression [53–55]. Polar flagella genes reside located on Chromosome 1, organized into two primary clusters—Region 1 (VP0770‐VP0791) and Region 2 (VP2224‐VP2261)—along with scattered genes (VP0689‐VP0690, VP2811, and VP2111) [52]. Lateral flagella genes are located on Chromosome 2, clustered in Region 1 (VP0061‐VPA0063 and VPA0264‐VPA0275) and Region 2 (VPA1532‐VPA1557) [52].

Deletion of *hns* markedly reduces *V. parahaemolyticus* swimming motility [14]. H‐NS directly promotes transcription of several polar flagellar operons, including *flgMN*, *flgAMN*, *flgBCDEFGHIJ*, and *flgKL-flaC* (Table 1) [14], thereby positively regulating swimming motility and polar flagellar gene expression. Conversely, H‐NS suppresses swarming motility by directly repressing *lafA* and *filD* (Table 1), which encode lateral flagellin and the filament cap, respectively [56, 57]. VpaH, an H‐NS homolog (54% identity to *V. parahaemolyticus* H‐NS), is uniquely present in *trh*‐positive strains [58]. Deletion of *vpaH* abolishes motility on semisolid plates and reduces lateral flagella biogenesis [58]. In summary, H‐NS promotes polar flagellar gene expression and swimming motility while repressing lateral flagellar genes and swarming motility in *V. parahaemolyticus*. However, the molecular basis for this dual regulatory role remains unclear. Key unresolved questions include (1) Why does H‐NS exert opposing effects on polar and lateral flagellar systems? (2) What physiological significance does this regulatory dichotomy hold? (3) How does H‐NS‐mediated regulation of motility integrate with the opposing effects of the QS master regulators AphA (activator) and OpaR (repressor)?

**TABLE 1:** Direct targets of H‐NS within polar and lateral flagellar genetic clusters.

Flagella	Gene	Locus tag	Operon	Function
Polar flagellum	*flgM*	VP0771	*flgMN*	anti‐σ^28^ factor
	*flgA*	VP0772	*flgAMN*	P‐ring chaperone/assembly
	*flgB*	VP0775	*flgBCDEFGHIJ*	Proximal rod
	*flgK*	VP0785	*flgKL-flaC*	HAP1 (hook‐filament junction)

Lateral flagella	*flaK*	VPA1548	—	Flagellin
	*fliD*	VPA1550	*fliDSTKLA-motAB*	Filament cap

### 2.5. Regulation of Biofilm Formation by H‐NS

Biofilms are structured microbial communities enclosed in a matrix on surfaces [4]. The biofilm matrix mainly consists of extracellular polysaccharides (EPS), proteins, nucleic acids (eDNA), and lipids, providing protection against harsh growth conditions and facilitating bacterial colonization [4]. Among these, EPS is the primary matrix constituent. High EPS production results in wrinkled colony morphology, while low EPS yields smooth colonies [59]. Two operons, *cpsA–K* and *scvA–O*, are linked to EPS biosynthesis in *V. parahaemolyticus*, though only *cpsA–K* directly governs the wrinkled phenotype [60]. Biofilm formation is a classic QS‐controlled phenotype, with AphA activating and OpaR generally repressing biofilm‐related genes [61, 62].

H‐NS regulates EPS production and biofilm formation in *V. parahaemolyticus* [63, 64]. The *hns* mutant exhibits significant defects in submerged biofilm formation compared to wild‐type strains [63]. However, deletion of *hns* leads to smooth colonies on agars, significantly decreases transcriptional levels of *cpsA-K*, and represses transcription of *scrABC*, which encodes a guanylate cyclase that degrades the second messenger molecule c‐di‐GMP [64]. This molecule promotes biofilm formation while inhibiting motility and virulence gene expression [65]. Thus, H‐NS appears to activate biofilm formation in *V. parahaemolyticus*. Contrastingly, a recent study shows that *hns* deletion enhances biofilm formation (as assessed by crystal violet staining) and c‐di‐GMP levels in *V. parahaemolyticus* [66]. H‐NS can bind upstream promoter–proximal regions of genes encoding c‐di‐GMP metabolism‐associated proteins, including VP0117, VP0699, *scrG*, VP2979, VPA0198, *scrA*, and VPA1176, thereby repressing c‐di‐GMP production [66]. Although H‐NS likely represses biofilm formation in *V. parahaemolyticus* by reducing c‐di‐GMP levels, the regulatory mechanisms require further investigation. The seemingly contradictory roles of H‐NS in biofilm regulation highlight the complexity of its function and its potential context‐dependent interaction with other regulators, including the QS system.

### 2.6. Regulation of Osmotic Stress Adaptation and Other Pathways by H‐NS

Beyond the core phenotypes of virulence, motility, and biofilm, H‐NS also regulates additional adaptive pathways in *V. parahaemolyticus*, highlighting its multifunctional role. A prominent example is osmotic stress adaptation. *V. parahaemolyticus* produces the compatible solute ectoine through the *ectABC-asp_ect* operon to cope with high osmolarity [67, 68]. This operon is also subject to complex regulation: it is oppositely controlled by the QS master regulator AphA and OpaR, with AphA activating and OpaR repressing its transcription. Additionally, H‐NS directly represses the *ectABC-asp_ect* operon, while the transcriptional regulator LeuO acts as an antisilencer that relieves H‐NS‐mediated repression, enabling operon expression during osmotic stress [67]. This illustrates a layered regulatory mechanism in which H‐NS silences stress‐response genes, a repression that can be reversed by specific antisilencers such as LeuO upon environmental stimulation. Such a model may apply to other H‐NS‐regulated loci. Future studies should examine the involvement of H‐NS in broader cellular processes—including general stress responses, central metabolism, and antimicrobial resistance—to fully delineate its regulatory scope.

### 2.7. A Proposed Integrative Model of H‐NS Function

**FIGURE 3:**
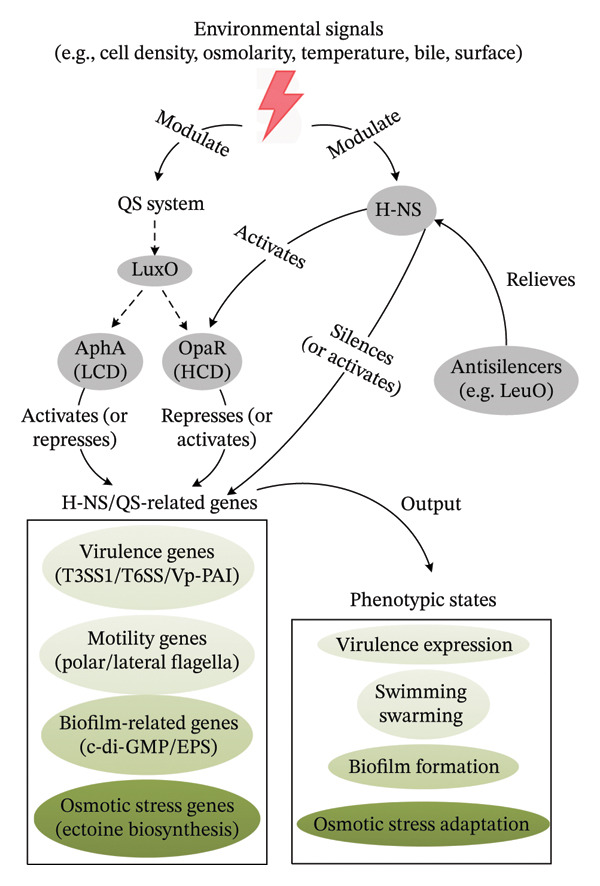
An integrative model of H‐NS‐mediated regulatory network in *V. parahaemolyticus*. Environmental signals—such as cell density, osmolarity, temperature, bile salts, and surface contact—are sensed by H‐NS or the QS system, which differentially activates the LCD master regulator AphA and the HCD master regulator OpaR. H‐NS serves as a global silencer of AT‐rich genomic regions, repressing genes involved in virulence, motility, biofilm formation, and osmotic stress adaptation. Its silencing activity is modulated by environmental cues and can be relieved by antisilencer proteins such as LeuO. The QS regulators AphA and OpaR further fine‐tune the expression of gene sets that are also under H‐NS control. The integrated output of this multilayered network determines the phenotypic state of the bacterium, including the expression of virulence factors, flagellar motility, biofilm formation, and osmotic stress adaptation.

To synthesize the diverse roles of H‐NS and its interplay with global regulators like QS, we propose an integrative model (Figure 3). In this model, environmental signals (e.g., cell density, osmolarity, temperature) influence the activity of master regulators like the QS system (AphA/OpaR) and specific sensors (e.g., for bile salts, surface contact). H‐NS acts as a pervasive genomic silencer, particularly of AT‐rich acquired genes, including many virulence factors (T3SS, T6SS) [10, 41], stress adaptation genes (*ectABC-asp_ect*) [67], and components of other pathways. The activity of H‐NS can be modulated by environmental conditions (e.g., salt, temperature) and by antisilencing proteins like LeuO [47, 67]. The QS system, through its master regulators AphA (LCD) and OpaR (HCD), provides a cell‐density overlay to this regulation, activating or repressing subsets of genes that are also under H‐NS control [13, 23]. H‐NS may influence the QS circuit itself (e.g., by activating *opaR*) [13]. The concerted and often antagonistic actions of H‐NS, QS regulators, and other specific factors ultimately determine the expression output for key phenotypes: virulence, motility (swimming vs. swarming), biofilm formation, and stress adaptation. This model positions H‐NS as a central integrator that fine‐tunes gene expression in response to multiple inputs, contributing to the ecological success and pathogenicity of *V. parahaemolyticus*.

## 3. Conclusion and Future Perspective

H‐NS is an abundant DNA‐binding protein that plays multiple functions, including facilitating genome evolution, modulating DNA folding, and global regulating gene regulation. Although H‐NS homologs from *E. coli* and *V. parahaemolyticus* exhibit low sequence conservation at the amino acid level, both share a conserved ‘QGR’ motif located between the β2 strand and H5 helix. This structural similarity suggests that these homologs may employ analogous DNA‐binding mechanisms to regulate gene expression. H‐NS plays multiple roles in *V. parahaemolyticus*, acting as a key node within the regulatory network. It not only regulates virulence gene expression, modulates bacterial motility, and participates in biofilm formation but also extends its influence to osmotic stress adaptation via the *ectABC-asp_ect* operon. However, many aspects of its function remain understudied: (1) while H‐NS acetylation is known to influence its activity in other systems [69], the specific acetylation sites and their functional consequences in *V. parahaemolyticus* remain uncharacterized; (2) H‐NS contributes to genomic stability in other bacteria, but its role in maintaining or regulating genome integrity in *V. parahaemolyticus* is unknown; (3) although H‐NS regulates virulence and biofilm‐related genes, potential functional synergies with other regulatory proteins like the QS master regulators AphA/OpaR, LeuO, and specific virulence regulators and their collective impact on gene expression networks are unresolved; (4) the precise mechanisms by which H‐NS modulates biofilm formation are poorly defined; (5) broader regulatory roles of H‐NS in other cellular pathways (e.g., stress response, metabolism, and resistance) remain largely unexplored. The proposed integrative model (Figure 3) provides a framework for understanding how H‐NS interfaces with global and specific signals to coordinate diverse phenotypes. In summary, H‐NS function in *V. parahaemolyticus* warrants deeper investigation, particularly regarding its posttranslational modifications, genomic interactions, partner proteins, and mechanistic contributions to biofilm dynamics and other adaptive responses.

## Author Contributions

Hao Tang drafted the manuscript. Yiquan Zhang commented on and suggested improvements to the manuscript.

## Funding

Work in our labs was supported by the Zhenjiang City Science and Technology Plan‐Basic Research Project (JC2025044) and the Natural Science Foundation of Nantong (JC2023045).

## Disclosure

All authors read and approved the final manuscript.

## Conflicts of Interest

The authors declare no conflicts of interest.

## Data Availability

All relevant data are within the manuscript.
